# Comparison of anticoagulation control and outcomes between usual medical care and pharmacist-led anticoagulation service in ambulatory patients taking warfarin at tertiary hospital in Ethiopia: a quasi-experimental study

**DOI:** 10.1186/s40780-024-00355-9

**Published:** 2024-06-26

**Authors:** Tamrat Assefa Tadesse, Amha Gebremedhin, Dejuma Yadeta, Legese Chelkeba, Teferi Gedif Fenta

**Affiliations:** 1 Department of Pharmaceutics and Social Pharmacy, School of Pharmacy, College of Health Sciences, Addis Ababa University, Addis Ababa, Ethiopia; 2 Department of Pharmacology and Clinical Pharmacy, School of Pharmacy, College of Health Sciences, Addis Ababa University, Addis Ababa, Ethiopia; 3 Department of Internal Medicine, School of Medicine, College of Health Sciences, Addis Ababa University, Addis Ababa, Ethiopia

**Keywords:** Oral anticoagulation, Pharmacist-led anticoagulation clinic, Usual medical care, Quasi-experimental study, Warfarin, Ethiopia

## Abstract

**Background:**

We aimed to compare anticoagulation control and outcomes between usual medical care (UMC) and pharmacist-led anticoagulation services (PLAS) in patients receiving warfarin at the Tikur Anbessa Specialized Hospital (TASH), Addis Ababa, Ethiopia.

**Methods:**

A quasi-experimental study was conducted, including 350 (66.7%) and 175 (33.3%) patients from the UMC and PLAS groups, respectively, from 525 patients. The time in therapeutic range (TTR) was determined using the Rosendaal method, with a TTR ≥ 65% set as the cut-off for optimal anticoagulation. The two-sample Wilcoxon rank-sum (Mann–Whitney U) test was used to compare continuous variables between groups. Categorical variables were compared between groups using Pearson’s chi-square test or Fisher’s exact test. Logistic regression and negative binomial regression analyses were conducted to identify the factors associated with suboptimal TTR and secondary outcomes, respectively, at the *p* values < 0.05, and 95% confidence interval (CI).

**Results:**

Compared with the UMC group, the patients in the PLAC group showed a significantly higher median (IQR) TTR [60.89% (43.5–74.69%) vs. 53.65% (33.92–69.14%), *p* < 0.001]. A significantly higher optimal TTR (≥ 65%) was achieved in the PLAC group (41.7% vs. 31.7%) than in the UMC group (*p* = 0.002). The odds of having a poor TTR were reduced by 43% (AOR = 0.57, 95% CI = 0.36–0.88, *p* = 0.01) among patients in the PLAC group compared to those in the UMC group. There were no statistically significant differences in the secondary outcomes between the groups, except for all-cause emergency visits (*p* = 0.003). The incidence of bleeding events decreased by 3% (IRR = 0.97, 95% CI = 0.96–0.99, *p* < 0.001) for every increase in INR monitoring frequency. The incidence of thromboembolic events increased by a factor of 15.13 (IRR = 15.13, 95% CI = 1.47–155.52, *p* = 0.02) among patients with a high-risk CHA_2_DS_2_-VASc score compared with those with a moderate score.

**Conclusion:**

Patients in the PLAC group had a significantly higher median TTR than those in the UMC group did. There were no statistically significant differences in the secondary outcomes between the groups, except for fewer all-cause emergency department visits in the PLAC group.

## Introduction

Warfarin is a widely used anticoagulant to prevent and treat thrombosis in various conditions, including venous thromboembolism (VTE), atrial fibrillation, post-myocardial infarction, and heart valve replacement [1–3]. Its narrow therapeutic range; frequent drug, herbal, and food interactions; and the effect of comorbidities place patients at risk of bleeding and thromboembolic complications if the recommended anticoagulation target ranges are not achieved [4–8]. Studies have shown that the effectiveness of warfarin therapy depends on the percentage of time that the international normalized ratio(INR) is within the therapeutic range or the time in the therapeutic range (TTR) [5, 9–12]. A minimum TTR of 65% is required for warfarin to be considered effective in the prevention and treatment of thromboembolic diseases [13, 14]. The risk of thromboembolism and bleeding has also been shown to depend on TTR [15], as evidenced by increased adverse clinical outcomes (ischemic stroke/transient ischemic attack, major bleeding, intracranial hemorrhage, and death) in patients with non-valvular atrial fibrillation in Thailand [16]. In addition, a systematic review and meta-regression analysis showed the importance of higher mean TTR, as it was significantly associated with a decreased rate of major bleeding and stroke/systemic embolism [17]. However, in clinical practice, maintaining adequate anticoagulation with warfarin has proven challenging, as demonstrated by studies conducted globally documenting a low TTR (< 65%) [18–28]. This problem appears to be the worst in Africa because the lowest TTRs have been reported in studies conducted in various regions of the continent [1, 8, 24, 29–32]. Lower TTRs (29–42.7%) were recorded in patients receiving warfarin in Ethiopia [33–37] Furthermore, a prevalence of 99.2% [38] and 21.1% ( [39] of warfarin-drug interactions were documented in two Ethiopian hospitals.

These findings highlight the need for alternative strategies to improve anticoagulant therapy [40, 41]. Specialized anticoagulation management services (AMS) have successfully optimized anticoagulation therapy to evaluate and monitor patients, provide ongoing patient education, and serve as a resource for both patients and physicians. Pharmacist-led anticoagulation service (PLAS) is becoming the best practice to achieve a better quality of care in patients receiving anticoagulants compared to other models. Increased TTR, decreased rates of admission and average clinic visits, lower risk of total bleeding and thrombosis events [42–44], and improved adherence to treatment have been documented in the pharmacist-led anticoagulation clinic (PLAC) group compared to the physician-led group [45, 46]. Furthermore, systematic reviews and meta-analyses have shown that pharmacist-led anticoagulation management results in lower rates of total bleeding and thrombotic events [42, 47], better quality of anticoagulation control, and lower healthcare utilization [47]. A retrospective cohort study in Malaysia revealed a significant association between the usual medical care (UMC) group and pharmacist-led warfarin medication therapy adherence clinics (WMTAC) in terms of TTR (*p* = 0.01), expanded therapeutic INR range (*p* < 0.04), and INR levels (*p* = 0.02) [48]. Another study conducted in Brazil reported significantly improved TTR values after pharmaceutical care in patients with AF with a low TTR (< 50%) [45, 49].

Given the practical limitations and complex nature of effective anticoagulant delivery, the adoption of such practices might improve the treatment response and patient outcomes in resource-constrained settings. Based on evidence reported elsewhere regarding the importance of PLAS in improving the quality of anticoagulation in patients receiving warfarin [42, 47, 48, 50–52] and needs assessment study recommendations in the same hospital [53], the first pharmacist-led anticoagulation clinic (PLAC) in Ethiopia was established at the Tikur Anbessa Specialized Hospital (TASH) in April 2018 under the cardiac clinic. The novelty of our study lies in the distinctive healthcare context of Ethiopia, which requires specific intervention strategies tailored to resource-constrained environments, culturally diverse settings, lower health literacy levels, limited diagnostic tools, underdeveloped electronic health records, and a shortage of well-trained healthcare providers. By focusing on these distinctive intervention strategies, our study was designed to evaluate the feasibility and effectiveness of PLAC in resource-limited settings by comparing anticoagulation control and outcomes between PLAC and UMC, hypothesizing improved anticoagulation control and outcomes in the PLAC group.

## Methods

### Study setting

This study was conducted at the cardiac and hematology clinics (CHCs) and PLAC of the Tikur Anbessa Specialized Hospital (TASH), a central referral hospital in Addis Ababa, Ethiopia. The CHCs operate four days per week, with an average of 600 patients per week. Cardiologists, hematologists, cardiac and hematology fellows, residents, physicians, and nurses staffed the clinics. PLAC was established at TASH in April 2018 as part of the anticoagulation management quality improvement process, and it is the only clinic in Ethiopia. The PLAC was staffed with four clinical pharmacists who provided AMS two days per week (Tuesday morning and Friday afternoon). It was established to serve patients with frequent nontherapeutic INRs, complicated anticoagulation history, and poor adherence to warfarin at the CHCs of the hospital. CHCs and PLAC were the clinics that mostly prescribed anticoagulants (mainly warfarin) for patients who had been followed up at the outpatient department of the hospital.

### Study design and period

A quasi-experimental study was conducted to evaluate the differences in anticoagulation control and outcomes between UMC and PLAS in adult patients receiving anticoagulation management services (AMS) with warfarin therapy at TASH from July 2021 to June 2023. The electronic database (iCare) of TASH was used to collect patient data related to clinical profiles and anticoagulation management-related information. A comprehensive Excel database was developed to systematically document and capture pertinent information regarding anticoagulation management during each visit for the PLAC group.

### Study protocol and intervention

Study participants who had been receiving AMS at the PLAC and CHCs in the hospital were grouped into an intervention group (PLAC) and a comparison group (UMC). Anticoagulation management services focus on optimizing anticoagulation control by managing patients receiving anticoagulants that require proper management to minimize serious adverse events, including excessive bleeding. The UMC group received existing AMS from physicians without the involvement of clinical pharmacists. The PLAC protocol, clinical judgment, and knowledge were used to develop a care plan for warfarin dose adjustment, follow-up INR testing, and patient counselling. Based on the hospital PLAC protocol recommendations, patients with at least two consecutive non-therapeutic INRs, complicated anticoagulation history, and recent warfarin non-adherence history at the UMC were assigned to the PLAC group and explained how they were enrolled and the services provided at the clinic. The protocol included detailed clinical information on the management of long-term warfarin therapy (indication, INR target ranges, duration of treatment, maintenance dosing algorithm (dosing adjustment recommendation), status of anticoagulation and action to be followed, frequency of INR monitoring, any warfarin-interacting drugs, and measures to be taken). The PLAC protocol was reviewed and approved by the anticoagulation team to provide adequate and appropriate AMS. Education (prepared in the Amharic language) that included all aspects of warfarin treatment was provided for approximately 20 min to all patients after enrolment in PLAC. The participants were provided with a follow-up booklet (a form with INR values, dosing scheme, next appointment date, and the most important messages provided during education). Furthermore, a medication review was performed to minimize drug interactions with warfarin. If necessary, the treating physicians communicated with a proposal for a drug change, dose adjustment, or another measure. For patients who did not visit the clinic per schedule or in cases of extreme non-therapeutic INRs (INR values < 1.5, or > 5) in which it was difficult to wait until the next clinic day to adjust the dose, phone consultation was used for appropriate actions and further evaluation. However, clinical pharmacists working in PLAC did not initiate warfarin for new patients and did not stop it; instead, they provided recommendations to the assigned physicians.

### Source and study population

The source population for this study included all outpatients who had follow-up at CHCs and PLAC of the TASH, whereas the study population consisted of patients who received AMS from these clinics, had been receiving warfarin therapy, and met the predefined study inclusion criteria.

### Eligibility criteria

In both groups, we enrolled patients aged ≥ 18 years who had received warfarin for at least six months and had a documented history of at least three consecutive INR measurements. This criterion was implemented to ensure the consistency and reliability of INR data, excluding individuals who were newly initiated on warfarin therapy, and to facilitate accurate calculation of TTR. Additionally, to qualify for inclusion in the PLAC cohort, patients were required to have initially received warfarin therapy at CHCs. Patients with interrupted INR monitoring or planned temporary interruptions, those who missed more than one consecutive clinic visit, those who received AMS from both PLAC and CHCs during the follow-up period, and those with incomplete or missing medical and medication records were excluded. These exclusion criteria were implemented to maintain the integrity of the study cohort and ensure the reliability of data analysis.

### Sample size determination, sampling technique, and participants’ recruitment

The sample size was calculated using the mean TTR of 42% from a recent study report at the Ethiopian public hospital [33], and the standard deviation of TTR was 0.25, based on the fact that anticoagulation control before implementing PLAS was better than that found in a previous study (0.42). It also assumed a Type I error (α-level) rate of 0.05, power of 80%, and UMC: PLAS ratio of 2:1. To achieve a minimum clinically important difference of 0.08 (i.e., an improvement from 42 to 50% in TTR), at least 304 patients in the control group (UMC) and 152 PLAS participants (including a 10% contingency for loss to follow-up) were required. Based on the determination and maintenance of the indicated eligibility criteria, we included 350 and 175 patients in the UMC and PLAS groups, respectively, in the final analysis. All patients who received AMS at CHCs, PLAC, and warfarin for at least six months prior to data collection were initially screened from clinical records. Eligible patients were recruited by a systematic random sampling technique using the formula (k = N/n), and every kth (actual sampling fraction) patient data were reviewed among patients who visited the clinics up to the sample size achieved. The actual sampling fraction (k) varied on different days as well as between the UMC and PLAC owing to variations in the total number of patients attending the clinics. The first study participant was selected by simple random sampling and every four–five and two patients were enrolled from the UMC and PLAC, respectively.

### Data collection instrument

A structured data collection tool was designed to collect all the required sociodemographic characteristics and clinical data. It included information on comorbidities, warfarin therapy, warfarin indications, daily warfarin dose, target INR ranges, INR values at each visit, interval days between INR monitoring, anticoagulation status at each visit (subtherapeutic, in target, and supratherapeutic ranges), measures taken by physicians and pharmacists, potential warfarin-interacting drugs, adverse drug events, any thromboembolism and bleeding events (both minor and major bleeding), emergency room visits, and hospitalizations between clinic visits. Stroke and bleeding risks were assessed at baseline in patients with atrial fibrillation using the CHA2DS2-Vasc score (Congestive heart failure, Hypertension, Age ≥ 75 years, Diabetes mellitus, Stroke, Vascular disease, Age 65–74 years, Sex category (female) = 0 in men, or 1 in women) and by the HAS-BLED (Hypertension, Abnormal renal/liver function, Stroke, Bleeding history or predisposition, Labile INR, Elderly (> 65 years), Drugs/alcohol) score to identify patients at high risk of bleeding (HAS-BLED score ≥ 3) [54].

### Study outcomes

The primary outcome was the percentage of median TTR differences between the two groups. TTR was determined using the Roosendaal Method, which determines TTR by incorporating INR measurement frequency and values, assuming that changes between consecutive INR measurements are linear [55]. In this study, a TTR ≥ 65% was used as the cutoff range for good/optimal anticoagulation control with warfarin therapy [13, 14]. Bleeding episodes, thromboembolic events, all-cause hospitalization, and emergency department visits differences between groups were the secondary outcomes of this study.

### Data collectors’ recruitment

Four clinical pharmacists who were selected based on their educational qualifications, clinical and research experience and familiarity with serving patients who required warfarin therapy collected data.

### Data quality assurance

The data collection instrument was reviewed and validated by a team of senior clinical pharmacists, cardiologists, and hematologists for its content, flow, completeness, and clarity to be used by data collectors and its suitability to the local context. A pre-test was conducted on 5% of the study population in both groups, and all necessary amendments and modifications were made to the structure before actual data collection. One-day training was provided to the data collectors on how to use the data collection instruments, study criteria/protocol, implementation of sampling techniques, maintenance of data confidentiality, and collection of patient data from the hospital’s electronic database (I-care system). Collected data were evaluated for completeness and consistency during data management, storage, and analysis.

### Data analysis

Data analysis was performed using the Statistical Package for the Social Sciences (SPSS) version 27. Descriptive statistics were used to summarize the demographic and clinical characteristics, CHA2DS2VASc score, HAS-BLED score, percentage of days with different therapeutic ranges, and the incidence of secondary outcomes. The two-sample Wilcoxon rank-sum (Mann–Whitney U) test was used to compare continuous variables between groups. Categorical variables were compared between groups using Pearson’s chi-square test or Fisher’s exact test. Binary logistic regression analysis was conducted to identify factors associated with poor TTR. Negative binomial regression analysis was performed to identify predictors of secondary outcomes, including bleeding episodes, thromboembolic events, all-cause emergency department visits, and hospitalization. Variables with a *p*-value < 0.25 in the bivariate analysis and binary negative binomial regression were included in the multivariable regression model and multivariable negative binomial regression. The significance of the association was determined at a 95% confidence level and a *p*-value < 0.05 both in the multivariable model and negative binomial regression analysis. The goodness-of-fit of the negative binomial regression model was evaluated using a likelihood ratio test by comparing fitted and null models.

### Ethical approval

Ethical approval was obtained from the Ethical Review Committee of the School of Pharmacy (ERB/SOP/454/2022) and Institutional Review Board (096/22/SoP) of the College of Health Sciences, Addis Ababa University, Ethiopia. Written permission to access the clinical data of the study participants was obtained from the outpatient department of the hospital. Since the PLAS at the TASH has been provided under the supervision of cardiologists and hematologists, any anticipated risk to patients has been communicated and linked to consultant cardiologists and hematologists.

## Results

### Socio-demographic characteristics of study participants

Of the 525 patients who participated in the study, 350 (66.7%) and 175 (33.3%) were included in the UMC and PLAC groups, respectively, and 375 (71.4%) were female. Most patients in both groups were female, with no significant differences (*p* = 0.31) between the groups in each sex category. The median (IQR) age in the UMC was 41.00 [32–55] years, with a range of 18–82 years, while in the PLAC group, the median ± IQR of age was 39.00 [32–49] years, with a range of 20–85 years without any significant difference between the groups (*p* = 0.15). A significant difference was observed based on the residence of the patients, and the majority of patients were residents of Addis Ababa (study area) in both the UMC and PLAC: 247 (70.6%) and 148 (84.6%), respectively (*p* < 0.001) (Table 1).

**Table 1:** Socio-demographic characteristics of patients receiving warfarin compared between UMC and PLAC at TASH

Variables	Total (*N* = 525) *n* (%)	UMC Group ( *N* = 350) *n* (%)	PLAC Group ( *N* = 175) *n* (%)	*P*-value
**Sex**				
Female	375(71.4)	245(70.0)	130(74.3)	0.31
Male	150(28.6)	105(30.0)	45(25.7)	
**Age in years**				
18–30	107(20.4)	72(20.6)	35(20.0)	0.46
31–45	231(44.0)	148(42.3)	83(47.4)	
46–64	135(25.7)	97(27.7)	38(21.7)	
≥ 65	52(9.9)	33(9.4)	19(10.9)	
Median (IQR) age	40.00 (32–53)	41(32–55)	39(32–49)	0.15
**Residence**				
Addis Ababa	395(75.2)	247(70.6)	148(84.6)	< 0.001
Out of Addis Ababa	130(24.8)	103(29.4)	27(15.4)	

### Clinical characteristics of patients

The clinical characteristics of the patients revealed a notable prevalence of underlying heart problems and comorbidities in both study groups, with a significantly higher incidence in the UMC group than in the PLAC group (82.3% vs. 64.6%, *p* < 0.001). The most prevalent clinical conditions in the UMC cohort were heart failure (45.4%) and chronic rheumatic valvular heart disease (CRVHD) (41.4%). Conversely, in the PLAC group, CRVHD (32.6%) was the predominant comorbidity, followed by heart failure (29.1%). Furthermore, notable differences (*p* < 0.05) in the presence of heart failure, hypertension, history of vascular disease, coronary artery disease/ischemic heart disease, and polycythemia vera and aspirin and/or clopidogrel use were observed between the two groups and were more frequently encountered in the UMC group than in the PLAC group, as shown in Table 2.

**Table 2:** Clinical characteristics of patients receiving warfarin compared between UMC and PLAC at TASH

Characteristics	Total ( *N* = 525) *n* (%)	UMC Group ( *N* = 350) *n* (%)	PLAC Group ( *N* = 175) *n* (%)	*P*-value
**Presence of heart problems or comorbidities**				
Yes	401(76.4)	288(82.3)	113(64.6)	< 0.001
**Underlying heart problems, comorbidities, or medication use**				
Heart failure	209(39.8)	158(45.4)	51(29.1)	< 0.001
Valvular heart disease	202(38.5)	145(41.4)	57(32.6)	0.06
Hypertension	109(20.8)	84(24.0)	25(14.3)	0.01
Stroke and thromboembolism history	88(16.8)	66(18.9)	22(12.6)	0.08
Amiodarone use	6 (1.1)	6 (1.7)	0 (0)	0.19
Aspirin, clopidogrel, NSAIDs use	31 (5.9)	26 (7.4)	5 (2.9)	0.04
Pulmonary Hypertension	63(12.0)	43(12.3)	20(11.4)	0.78
Vascular heart disease history^b^	43(8.2)	39(11.1)	4(2.3)	< 0.001
Diabetes Mellitus	35(6.7)	28(8.0)	7(4.0)	0.08
Neurologic disorders^c^	22(4.2)	19(5.4)	3(1.7)	0.06
CAD/IHD without thrombus	20(3.8)	18(5.1)	2(1.1)	0.02
Seizure disorders	20(3.8)	17(4.9)	3(1.7)	0.09
Cardiomyopathy	23(4.4)	17(4.9)	6(3.4)	0.51
HIV/AIDS	14(2.7)	12(3.4)	2(1.1)	0.16
Cancer	13(2.5)	12(3.4)	1(0.6)	0.07
Hypertensive heart disease	20(3.8)	11(3.1)	9(5.1)	0.33
Hyperthyroidism	14(2.7)	10(2.9)	4(2.3)	0.78
Chronic Pulmonary diseases^d^	14(2.7)	10(2.9)	4(2.3)	0.78
Dyslipidemia	11(2.1)	10(2.9)	1(0.6)	0.11
Rheumatologic diseases	13(2.5)	9(2.6)	4(2.3)	0.55
Gastric illness/peptic ulcer diseases	10(1.9)	9(2.6)	1(0.6)	0.18
Portal hypertension	7(1.3)	7(2.0)	0(0.0)	0.10
Renal Diseases	12(2.3)	8(2.3)	4(2.3)	0.63
Liver diseases	11(2.1)	8(2.3)	3(1.7)	0.76
Polycythemia vera	8(1.5)	8(2.3)	0(0)	< 0.001
Iron deficiency anemia	7(1.3)	6(1.7)	1(0.6)	0.43
Hypothyroidism	6(1.1)	4(1.1)	2(1.1)	0.68
Psychiatric disorders	5(0.9)	3(0.9)	2(1.1)	0.54
Others^e^	27(5.1)	18(5.1)	9(5.1)	0.59

^a^includes transient ischemic attack (TIA), subarachnoid hemorrhage (SAH), arteriovenous malformations (AVM), and intracranial hemorrhage (ICH)

^b^incudes prior, myocardial infarction, peripheral artery disease, or aortic plaque

^c^includes hemiplegia, peripheral neuropathy, Parkinson’s disease, and chronic lower back pain

^d^includes COPD, Asthma, etc.; HIV/AIDS, nonsteroidal anti-inflammatory drugs; CAD/IHD, coronary artery disease/ischemic heart disease; and gynecological disorders, benign prosthetic hyperplasia, pituitary microadema, erectile dysfunction, tuberculosis, Infective Endocarditis, myoma, and visual impairment

Among the 210 patients in the UMC group and 125 patients in the PLAC group diagnosed with atrial fibrillation, 39.5% of those in the UMC group had a high CHA2DS2-VASc score, while 45.6% of patients in the PLAC group had a low CHA2DS2-VASc score. This discrepancy was statistically significant (*P* < 0.001). In contrast, the majority of patients in both cohorts presented with a moderate bleeding risk, constituting 71.4% of the UMC group and 54.4% of the PLAC group, with statistically significant differences between the groups (*p* < 0.001) (Table 3).

**Table 3:** Baseline stroke and bleeding risk score among atrial fibrillation patients receiving warfarin compared between UMC and PLAC at TASH

Risk score	Total (*N* = 335) *n* (%)	UMC Group (*N* = 210) *n* (%)	PLAC Group (*N* = 125) *n* (%)	*P*-value
**CHA**_**2**_**DS**_**2**_**-VASc Risk**				
Low	110(32.8)	53(25.2)	57(45.6)	
Moderate	120(35.8)	74(35.3)	46(36.8)	< 0.001
High	105(31.4)	83(39.5)	22(17.6)	
**HAS-BLED Risk**				
Low	80(23.8)	34(16.2)	46(36.8)	
Moderate	218(65.1)	150(71.4)	68(54.4)	< 0.001
High	37(11.1)	26(12.4)	11(8.8)	

A statistically significant difference in warfarin indication distribution among the groups was observed for atrial fibrillation (*p* = 0.01), mechanical heart valves (*p* = 0.002), bioprosthetic valve replacement/repair (*p* = 0.002), and post-percutaneous mitral balloon valvotomy (*p* = 0.01), as was most commonly indicated for the PLAC group. Patients with deep vein thrombosis (*p* < 0.001) and portal vein thrombosis (*p* = 0.019) were managed only at the UMC (Table 4).

**Table 4:** Warfarin indication among outpatients compared between UMC and PLAC

**Indication of warfarin**	**Total (*N* = 525)** ***n***(%)	**UMC Group** **(*N*** = 350) ***n*** %)	**PLAC Group** **(*N*** = 175) ***n*** (%)	***P***-value
Atrial fibrillation	335 (63.8)	210 (60.0)	125 (71.4)	0.01
Valvular heart disease	59 (11.2)	46 (13.1)	13 (7.4)	0.05
Cardioembolism^a^	49 (9.3)	33 (9.4)	16 (9.1)	0.92
Post heart valves (mechanical)^c^	84 (16.0)	44 (12.6)	40 (22.9)	0.002
Cardiac Thrombus^b^	18 (3.4)	10 (2.9)	8 (4.6)	0.31
(Bio) prosthetic valve replacement/ repair^c^	22 (4.2)	8 (2.3)	14 (8.0)	0.002
Post-percutaneous mitral balloon valvotomy^c^	27 (5.1)	12 (3.4)	15 (8.6)	0.012
Cardiomyopathy^c^	10 (1.9)	4 (1.1)	6 (3.4)	0.091
IHD with thrombus	10 (1.9)	4 (1.1)	6 (3.4)	0.091
Deep vein thrombosis	37 (7.0)	37 (10.6)	0 (0)	< 0.001
Pulmonary embolism	7 (2.0)	7 (2.0)	0 (0)	0.102
Portal vein thrombosis	11 (2.1)	11 (3.1)	0 (0)	0.019

^a^includes cardioembolic stroke, peripheral artery embolism), other site embolism), or non-embolic stroke (ischemic stroke)

^b^includes left ventricular/apical/arterial thrombus

^c^By themselves are not indications for warfarin; patients should have atrial fibrillation, high CHA2DS2-VASc, cardioembolism, cavity thrombus, or ventricular thrombus

### INR target range, monitoring frequency, and dose of warfarin

The majority of the patients in both groups had an INR target range of 2.0–3.0, with a statistically significant difference between the groups (*p* = 0.002). The INR was monitored at median intervals of 51.54 (IQR 37.18–69.14) days for the UMC group and 40.08 (IQR 33.17–48.83) days in the PLAC group, with a notable difference between the two cohorts (*p* < 0.001). The median weekly warfarin dose used in the PLAC group was 35 (27–42.5) mg, while in the UMC group, it was 32.26 (25.21- 40.56) mg without a statistically significant difference between the groups (*p* = 0.239). Similarly, there were no significant differences in the median prescribed warfarin dose between the two groups, as depicted in Table 5).

**Table 5:** INR target range, monitoring frequency, and dose of warfarin compared between UMC and PLAC at TASH

**Item description**	**Total (*N* = 525)** ***n*** (%)	**UMC Group** **(*N*** = 350) ***n*** %)	**PLAC Group** **(*N*** = 175) *n* (%)	***P***-value
**Target INR range**				
2.0–3.0	441(84.0)	306 (87.4)	135 (77.1)	0.002
2.5–3.5	84 (16.0)	44 (12.6)	40 (29.9)	
**INR monitoring frequency in days**				
Median (IQR)	46.45 (36.15–61.6)	51.54 (37.18–69.14)	40.08(33.17–48.83)	< 0.001
**Number of Visits**				
Median (IQR)	9 (7–9)	10 (7–13)	9 (7–11)	0.044
**Average Weekly Dose**				
Median (IQR)	33.22 (25.9–41.33)	32.36 (25.21–40.56)	35 (27–42.5)	0.239
**Average daily Dose**				
Median (IQR)	4.75(3.7–5.9)	4.62 (3.6–5.80)	5.00 (3.86–6.07)	0.239

### Differences in the time spent in different INR ranges

**Fig. 1:**
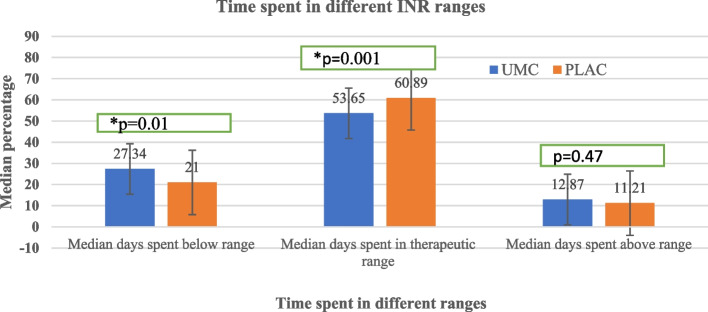
Difference in the time spent in different INR ranges between UMC and PLAC at TASH

Compared with the UMC group, patients in the PLAC group showed a significantly higher percentage of median (IQR) TTR 60.89% (43.5–74.69%) vs. 53.65% (33.92–69.14%), *p* < 0.001]. The percentage of time below range (TBR) was significantly lower in the PLAC group (21%, (8.5–37.78%) than in the UMC group (*p* = 0.01). Further analysis using the *R*-value (rank-biserial correlation coefficient) revealed that the effect size associated with the difference between UMC and PLAC, as measured by r, was 0.02. This indicated a 2% effect size, favoring PLAC (Fig. 1).

A significantly higher optimal TTR (≥ 65%) was achieved in the PLAC group (41.7% vs. 31.7%) than in the UMC group (*P* = 0.02) (Table 6).

**Table 6:** Comparison of optimal TTR difference between UMC and PLAC

Group	TTR < 65%		TTR ≥ 65%		*P* value
	*N* (%)	95%CI	*n* (%)	95% CI	
UMC	239(68.3)	(63.1,73.1)	111(31.7)	(26.9,36.9)	0.02
PLAC	102(58.3)	(50.6,65.7)	73 (41.7)	(34.3,49.4)	

### Predictors of poor the time in therapeutic range

The results of the multivariate logistic regression analysis showed that the odds of having a poor TTR were reduced by 43% (AOR = 0.57, 95% CI = 0.36–0.88, *p* = 0.01) among patients in the PLAC group compared to those in the UMC group. Patients with low HAS-BLED scores were 49% (AOR = 0.51, 95% CI = 0.29- 0.91, *p* = 0.02) less likely to have a poor TTR than those with no risk. Patients with moderate and high HAS-BLED scores were two and three times more likely to have a poor TTR (AOR = 1.68, 95% CI = 1.07–2.64, *p* = 0.03) and (AOR = 3.08, 95% CI = 1.17–8.09, *p* = 0.02), respectively, than patients with no risk. Additionally, patients with mechanical heart valves were twice as likely to have poor TTR (AOR = 2.11, 95% CI = 1.17–3.81 *p* = 0.01). The odds of poor TTR were reduced by 2% for every increase in the frequency of INR monitoring (AOR = 0.98, 95% CI = 0.97–0.99, *p* = 0.002) (Table 7).

**Table 7:** Factors associated with poor TTR among patients receiving warfarin therapy

Variable	TTR ≥ 65%	TTR < 65%	COR (95%CI)	AOR (95%CI)	*P*-value
**Treatment group**					
UMC	111(21.1)	239(45.5)	1.00	1.00	0.01*
PLAC	73(13.9)	102(19.4)	0.65(0.45,0.95)	0.57(0.36,0.88)	
**Sex**					
Female	132(25.1)	243(46.3)	1.00	1.00	0.79
Male	52(9.9)	98(18.7)	1.02(0.69,1.52)	0.95(0.62,1.44)	
**Age in years**					
18–30	32(6.1)	75(14.3)	1.00	1.00	
31–45	81(15.4)	150(28.6)	0.79(0.48,1.29)	0.79(0.47,1.32)	0.36
46–64	53(10.1)	82(15.6)	0.66(0.39,1.13)	0.57(0.32,1.02)	0.06
≥ 65	18(3.4)	34(6.5)	0.81(0.39,1.63)	0.45(0.19,1.03)	0.06
**HAS-BLED risk scores**					
No risk	65(12.4)	125(23.8)	1.00	1.00	
Low risk	46(8.8)	34(6.5)	0.38(0.23, 0.66)	0.51(0.29,0.91)	0.02*
Moderate risk	65(12.4)	153(29.1)	1.22(0.81,1.86)	1.68(1.07,2.64)	0.03*
High risk	8(1.5)	29(5.5)	1.89(0.82,4.36)	3.08(1.17,8.09)	0.02*
**Post-mechanical heart valves**					
No	163(31.0)	278(53.0)	1.00	1.00	0.01*
Yes	21(4.0)	63(12.0)	1.76(1.04,2.99)	2.11(1.17,3.81)	
**Post PMTC valvotomy**					
No	169(32.2)	329(62.7)	1.00	1.00	0.09
Yes	15(2.9)	12(2.3)	0.41(0.19,0.89)	0.49(0.22,1.13)	
**INR monitoring interval**			0.99(0.98,1.01)	0.98(0.97,0.99)	0.002*

### Secondary outcomes

Eighty-nine (25.4%) bleeding events were reported in the UMC group, with the highest record of eight bleeding episodes in a single patient. Forty-nine bleeding episodes occurred in the different patients. The remaining bleeding episodes were reported more than once during the different patient visits. In the PLAC group, 29 (16.6%) bleeding episodes occurred; four and one episodes were recorded in two and three patients, respectively, and the remaining 18 were recorded in different patients. There were no differences in bleeding events between the groups (*p* = 0.715). All bleeding events were minor, including bleeding from the nose, gums, and teeth, bruising, and menstruation. Fifteen (4.3%) and eight (4.6%) thromboembolic events were recorded in the UMC and PLAC groups, respectively, with no significant difference between the groups (*p* = 0.435). All-cause emergency department visits were documented in 64 (18.3%) and five (2.9%) patients in the UMC and PLAC groups, respectively, with a statistically significant difference between the groups (*p* = 0.003) (Table 8).

**Table 8:** Secondary outcomes among patients receiving warfarin compared between UMC and PLAC

**Secondary outcomes**	**Total** *n* (%)	**UMC** *n* (%)	**PLAC** *n* (%)	***p*-value**
Bleeding episodes	118(22.5)	89(25.4)	29(16.6)	0.715
Thromboembolic episodes	23(4.4)	15(4.3)	8(4.6)	0.435
Emergency department visits	69(13.1)	64(18.3)	5(2.9)	0.003
Hospitalization	76(14.5)	62(17.7)	14(8.0)	0.469

### Predictors of secondary outcomes

Negative binomial regression showed that the incidence of bleeding events decreased by 3% (IRR = 0.97, 95% CI = 0.96–0.99, *p* < 0.001) for every increase in INR monitoring frequency. The incidence of thromboembolic events increased by a factor of 15.13 (IRR = 15.13, 95% CI = 1.47–155.52, *p* = 0.02) among patients who had a high-risk CHA_2_DS_2_-VASc score compared to those with a moderate score. The incidence of all-emergency department visits was increased by a factor of 7.59 (IRR = 7.59, 95% CI = 2.68- 21.50, *p* < 0.001) in the UMC group compared to that in the PLAC group. All-emergency department visits were reduced by 69% (IRR = 0.31, 95% CI = 0.13–0.73, *p* = 0.007) in patients with a TTR < 65% compared to those with a TTR ≥ 65%. For every increase in the frequency of INR monitoring, the incidence of all-emergency department visits was reduced by 3% (IRR = 0.97, 95% CI = 0.95–0.99, *p* < 0.001). The incidence of hospitalization was reduced by 73% (IRR = 0.27, 95% CI = 0.10- 0.70, *p* = 0.007) in patients with a TTR < 65% compared with those with a TTR ≥ 65% (Table 9).

**Table 9:** Poisson regression analysis of secondary outcomes among patients receiving warfarin therapy

	**Bleeding episodes**		**Thromboembolic events**		**Emergency department visit**		**Hospitalization**	
**Variable**	**IRR (95%CI)**	***p*-value**	**IRR (95%CI)**	***p*-value**	**IRR (95%CI)**	***p*-value**	**IRR (95%CI)**	***p*-value**
**Patient group**								
UMC	1.45(0.79,2.65)	0.22	0.84(0.26,2.68)	0.77	7.59(2.68,21.50)	< 0.001*	1.75(0.79,3.87)	0.17
PLAC	1.00		1.00		1.00		1.00	
**Sex**								
Female	0.90(0.53,1.53)	0.69	0.55(0.21,1.45)	0.23	1.50(0.79,2.84)	0.21	0.82(0.43,1.57)	0.55
Male	1.00		1.00		1.00		1.00	
**Age**								
18–30	0.74(0.26,2.16)	0.58	0.85(0.13,5.53)	0.86	2.02(0.72,5.69)	0.19	1.31(0.39,4.39)	0.67
31–45	1.12(0.43,2.91)	0.82	1.07(0.21,5.46)	0.94	0.84(0.31,2.28)	0.73	1.00(0.33,3.02)	1.00
46–64	0.78(0.31,1.95)	0.59	0.69(0.13,3.66)	0.66	0.61(0.22,1.67)	0.34	0.51(0.17,1.56)	0.24
≥ 65	1.00		1.00		1.00		1.00	
**Residence**								
Addis Ababa	1.04(0.59,1.84)	0.89	1.25(0.41,3.84)	0.69	1.16(0.65,2.07)	0.62	1.57(0.77,3.17)	0.21
Out of Addis Ababa	1.00		1.00		1.00		1.00	
**Presence of comorbidities**								
No	1.65(0.56,4.87)	0.37	1.73(0.26,11.31)	0.57	0.69(0.22,2.25)	0.55	0.75(0.18,3.04)	0.68
Yes	1.00		1.00		1.00		1.00	
**CHA**_**2**_**DS**_**2**_**-VASc Risk**								
High risk	1.87(0.78,4.52)	0.16	15.13(1.47,155.52)	0.02*	1.79(0.80,4.03)	0.15	2.22(0.80,6.14)	0.13
Low risk	1.59(0.67,3.77)	0.29	4.09(0.44,38.07)	0.22	0.38(0.14,1.08)	0.06	1.11(0.37,3.32)	0.85
Moderate risk	1.00		1.00		1.00		1.00	
**HAS-BLED risk**								
High risk	0.81(0.27,2.39)	0.70	0.14(0.01,1.93)	0.14	0.44(0.14,1.44)	0.18	0.63(0.17,2.24)	0.47
Low risk	0.69(0.27,1.77)	0.44	1.56(0.29,8.08)	0.59	2.27(0.79,6.53)	0.13	1.09(0.35,3.41)	0.88
Moderate risk	1.00		1.00		1.0		1.00	
**Target INR range**								
2–3	0.87(0.42,1.78)	0.69	0.86(0.25,3.01)	0.81	1.06(0.41,2.79)	0.90	0.87(0.35,2.17)	0.76
2.5–3.5	1.00		1.00		1.00		1.00	
**Presence of warfarin drug interaction**								
No	0.99(0.57,1.72)	0.97	0.69(0.24,2.04)	0.51	0.90(0.49,1.62)	0.73	0.92(0.45,1.86)	0.81
Yes	1.00		1.00		1.00		1.00	
**Time in the therapeutic range**								
< 65%	0.86(0.39,1.88)	0.71	0.35(0.08,1.53)	0.16	0.31(0.13,0.73)	0.007*	0.27(0.10,0.70)	0.007*
≥ 65	1.00		1.00		1.0		1.00	
**Comorbidity score**								
No	0.22(0.05,0.97)	0.05	0.38(0.02,6.33)	0.49	0.54(0.12,2.38)	0.41	0.33(0.05,2.11)	0.24
Mild	0.43(0.16,1.17)	0.09	0.81(0.12,5.58)	0.83	0.99(0.37,2.71)	0.99	0.70(0.21,2.38)	0.57
Moderate	0.63(0.24,1.63)	0.34	0.31(0.04,2.53)	0.28	0.93(0.35,2.45)	0.88	1.18(0.36,3.85)	0.78
Severe	1.00		1.00					
\ INR monitoring interval	0.97(0.96,0.99)	< 0.001*	0.98(0.96,1.01)	0.17	0.97(0.95,0.99)	< 0.001*	0.98(0.97,1.00)	0.06
**Percentage of days in different INR ranges**								
Time below range	1.02(0.99,1.04)	0.07	0.99(0.93,1.07)	0.99	0.99(0.90,1.01)	0.83	0.99(0.95,1.03)	0.55
Time therapeutic range	1.01(0.99,1.04)	0.33	0.98(0.91,1.05)	0.53	0.97(0.88,1.06)	0.48	0.97(0.93,1.02)	0.19
Time above range	1.01(0.99,1.02)	0.38	0.99(0.92,1.06)	0.75	0.99(0.90,1.09)	0.83	1.00(0.97,1.04)	0.88
Average weekly warfarin dose	0.99(0.98,1.01)	0.93	0.99(0.96,1.02)	0.54	0.99(0.98,1.01)	0.71	0.99(0.98,1.02)	0.82

## Discussion

This study aimed to compare the effectiveness of UMC and PLAC in terms of anticoagulation control and outcome in patients receiving warfarin. It also provides crucial insights into optimizing warfarin therapy in resource-limited healthcare settings by highlighting the significant role pharmacists can play in enhancing anticoagulation management. Moreover, we identified factors associated with these outcomes (time in the therapeutic range and secondary outcomes, including bleeding, thromboembolic events, all-cause emergency department visits, and hospitalization). Although most patients in both groups were female and the UMC group experienced noticeably higher disease burdens, significant associations of sex and patient background differences with study outcomes were not found in our study.

In the present study, a significantly higher percentage of the median TTR was found in the PLAC group than in the UMC group (*p* < 0.001). This outcome aligns with previous studies demonstrating the superiority of PLAC over UMC in achieving a good TTR [9, 49, 56–58]. Moreover, a systematic review by Manzoor et al. [47] indicated that the majority of the included studies (83.0%) reported a statistically significant TTR in the pharmacist-managed group compared to routine medical care [47]. Another systematic review and meta-analysis also documented the superiority of PLAS over UMC in managing warfarin therapy based on observational studies, with a significantly higher TTR in the pharmacist-managed group [42, 59]. Furthermore, in this study, the proportion of patients who achieved optimal anticoagulation control (TTR ≥ 65%) was significantly higher in the PLAC group than in the UMC group (41.7% vs. 31.7%, *p* < 0.002), which is consistent with other reports that a higher proportion of patients in the pharmacist-led group reached the target anticoagulation physician-led clinic [9, 58]. These findings emphasize the potential of integrating pharmacist-led services into AMS, indicating a novel approach to improve anticoagulation control and outcomes in patients receiving warfarin therapy [60]. By comparing UMC with PLAS, the current study provides convincing evidence of the tangible benefits of pharmacist involvement in managing patients taking warfarin, offering a significant contribution to the evolving discourse on enhancing chronic disease management in limited-resource settings such as Ethiopia.

The effectiveness of PLAS not only emphasizes the potential of specialized anticoagulation services within the African healthcare landscape [50], but also aligns with global trends of advocating for enhanced warfarin therapy management [42]. Despite facing multifaceted challenges, including resource constraints, the successful implementation of PLAS in Ethiopia reflects the importance of providing quality AMS to optimize anticoagulation control and outcomes that resonate with a broader global shift towards the incorporation of pharmacist-led interventions in chronic disease management. This approach has been increasingly recognized for its capacity to provide personalized patient education, ensure consistent monitoring, and facilitate precise dosage adjustments, including the management of complex therapies, such as warfarin [61]. The difference between the outcomes of this study and the typical lower TTRs reported in African countries [32] further emphasizes the unique impact of PLAS in resource-limited settings. This finding suggests that PLAS may expand beyond high-income countries and offer a viable solution for improving anticoagulation management. This study noted more frequent INR monitoring in the PLAC group (40.08 median days) than in the UMC group (51.54 median days) (*p* < 0.001), which is an integral component for achieving better anticoagulation control in patients receiving warfarin therapy. Existing studies have shown the necessity for regular monitoring and timely dosage adjustments to maintain patients within their therapeutic range [62–64], which might have contributed to the observed improvement in TTR within the PLAC group in our study.

In the present study, the incidence of all the secondary outcomes was higher in the UMC group. However, a statistically significant difference was observed only in all-cause emergency visits (*p* = 0.003) between the two groups, and Entezari-Maleki et al. (2016) also reported fewer emergency department visits in the pharmacist-led group (*p* < 0 0.0001) [59]. The absence of significant differences between pharmacist-led interventions and other anticoagulation models in the incidence of bleeding and thromboembolic events has also been reported in two Chinese studies [56, 65]. However, other observational studies and systematic reviews have reported a significantly lower incidence of bleeding and thromboembolic events in pharmacist-warfarin therapy management [47, 59]. The observed lower incidence of emergency department visits among patients in the PLAC group in this study may highlight the proactive nature of PLAS in mitigating complications that would typically necessitate emergency intervention.

We identified several factors associated with poor TTR and incidence of secondary outcomes among patients receiving warfarin therapy. Multivariate regression analysis showed that intervention (PLAC) (AOR = 0.57, 95% CI = 0.36–0.88, *p* = 0.01) was an independent protective factor for poor anticoagulation quality (TTR < 65%). This finding aligns with previous studies conducted in China [65] and elsewhere [49, 66], which reported PLAS as an important intervention to reduce poor TTR in patients receiving anticoagulation therapy. The analysis also highlighted the relationship between an increased HAS-BLED score and poor TTR, emphasizing the vital need to evaluate the bleeding risk in anticoagulation management planning. This principle is widely acknowledged in international anticoagulation management guidelines, which advocate a balanced approach that considers both the therapeutic benefits and potential bleeding risks associated with warfarin therapy [54].

In the current study, we also found that the odds of poor TTR decreased with every increase in the INR monitoring frequency. Qiu et al. [65] reported this effect in other ways, that is, increasing the average interval of INR monitoring as an independent risk factor for poor anticoagulation quality [65]. However, Papala et al. (2021) showed that an increase in the INR testing interval length did not significantly decrease the overall mean clinical TTR [67]. Frequent INR monitoring with appropriate dose adjustments may contribute to better optimized anticoagulation control of PLAC. Moreover, patients with mechanical heart valves were more likely to have a poor TTR (AOR, 1.76; *p* = 0.01). This signifies the need for intensified monitoring with potentially more aggressive anticoagulation measures in these patient groups by implementing individualized care strategies and addressing the specific needs and higher risk of thromboembolic complications in patients with mechanical heart valves [68].

One of the findings of our study was the association between frequent INR monitoring and a notable reduction in the incidence of bleeding events and emergency department visits. This observation highlights the indispensable role of close monitoring in optimizing anticoagulation outcomes and reducing the incidence of warfarin-related complications. More frequent INR monitoring should be performed to improve patient safety and therapeutic efficacy, more-frequent INR monitoring should be performed [64]. Patients with higher CHA2DS2-VASc scores were at increased risk of thromboembolic events. This is consistent with a previous study that demonstrated that the CHA_2_DS_2_-VASc score is associated with thromboembolism [69].

The implications of this study on the management of warfarin therapy using PLAS in Ethiopia are profound and multifaceted. This supports the pivotal integration of pharmacists as essential contributors to the management of complex medication therapies, including warfarin, and highlights the necessity for health policymakers to advocate for the expansion of PLAS-type models in Ethiopian health systems. This approach is instrumental in enhancing the clinical outcomes of patients taking warfarin, particularly in resource-constrained settings such as Ethiopia, where warfarin is the drug of choice for the prevention and treatment of thrombosis in many patients. The success of PLAS in improving TTR and reducing complications related to warfarin therapy highlights the importance of incorporating pharmacists into multidisciplinary care teams, emphasizing their expertise in medication management and patient education. The potential benefits of such services within the Ethiopian healthcare system emphasize the universal applicability and efficacy of pharmacist-led care models in enhancing the management of warfarin therapy, and by extension, chronic disease management.

### Strengths and limitations of the study

This is the first study to evaluate the effects of PLAS on anticoagulation control and outcomes by comparing PLAS with UMC in Ethiopian patients undergoing warfarin therapy. The present study was unique in that it used an anticoagulation management protocol that consisted of a package of measures, including a warfarin-dosing algorithm, adherence support, and patient education regarding many aspects of warfarin therapy. This finding may serve as input for expanding PLAS to other Ethiopian health facilities and developing countries with similar health systems. However, it is essential to acknowledge the limitations of the present study. First, the execution of the study in a single healthcare center may limit its generalizability. This study was implemented in a hospital setting in Addis Ababa, Ethiopia; hence, the feasibility and effectiveness of PLAS may vary in other healthcare settings with different resources, patient populations, and organizational structures, thereby limiting the generalizability of the study findings. There could be inconsistencies among clinical pharmacists and physicians in their practices based on their educational backgrounds, which could affect the consistency and effectiveness of the intervention and require further consideration. This study did not compare the differences between the two anticoagulation models in terms of patients’ knowledge, satisfaction, and adherence to warfarin therapy, and we did not evaluate the economic implications of PLAS compared to UMC.

## Conclusion

This study concluded that patients in the PLAC group had a significantly higher median TTR than those in the UMC group did. A higher proportion of patients in the PLAC group achieved optimal TTR than those in the UMC group. The likelihood of poor TTR was lower in the PLAC group than in the UMC group. There were no statistically significant differences in the secondary outcomes between the groups, except for all-cause emergency department visits. This study substantially contributes to the argument that PLAS represents a crucial development in the management of patients taking warfarin to improve clinical outcomes and healthcare efficiency. A cost-effectiveness analysis is essential for evaluating the economic feasibility of incorporating PLAS into the Ethiopian healthcare system.

## Data Availability

Data is available upon request to the corresponding author.
