# Publisher Correction: Comparison of anticoagulation control and outcomes between usual medical care and pharmacist-led anticoagulation service in ambulatory patients taking warfarin at tertiary hospital in Ethiopia: a quasi-experimental study

**DOI:** 10.1186/s40780-024-00364-8

**Published:** 2024-07-19

**Authors:** Tamrat Assefa Tadesse, Amha Gebremedhin, Dejuma Yadeta, Legese Chelkeba, Teferi Gedif Fenta

**Affiliations:** 1 Department of Pharmaceutics and Social Pharmacy, School of Pharmacy, College of Health Sciences, Addis Ababa University, Addis Ababa, Ethiopia; 2 Department of Pharmacology and Clinical Pharmacy, School of Pharmacy, College of Health Sciences, Addis Ababa University, Addis Ababa, Ethiopia; 3 Department of Internal Medicine, School of Medicine, College of Health Sciences, Addis Ababa University, Addis Ababa, Ethiopia

**Publisher Correction: Journal of Pharmaceutical Health Care and Sciences (2024) 10:32**

10.1186/s40780-024-00355-9

In the original publication of this article an error occurred during the publication process.

In table 3 the heading stated “i=210” , the correct information is “N=210”. The original article has been updated. The publisher apologizes for the inconvenience caused to the authors & readers.

